# Cell Cycle Regulation and Apoptotic Responses of the Embryonic Chick Retina by Ionizing Radiation

**DOI:** 10.1371/journal.pone.0155093

**Published:** 2016-05-10

**Authors:** Margot Mayer, Nicole Kaiser, Paul G. Layer, Florian Frohns

**Affiliations:** Developmental Biology and Neurogenetics, Darmstadt University of Technology, Darmstadt, Germany; National Cancer Institute, UNITED STATES

## Abstract

Ionizing radiation (IR) exerts deleterious effects on the developing brain, since proliferative neuronal progenitor cells are highly sensitive to IR-induced DNA damage. Assuming a radiation response that is comparable to mammals, the chick embryo would represent a lower vertebrate model system that allows analysis of the mechanisms underlying this sensitivity, thereby contributing to the reduction, refinement and replacement of animal experiments. Thus, this study aimed to elucidate the radiation response of the embryonic chick retina in three selected embryonic stages. Our studies reveal a lack in the radiation-induced activation of a G1/S checkpoint, but rapid abrogation of G2/M progression after IR in retinal progenitors throughout development. Unlike cell cycle control, radiation-induced apoptosis (RIA) showed strong variations between its extent, dose dependency and temporal occurrence. Whereas the general sensitivity towards RIA declined with ongoing differentiation, its dose dependency constantly increased with age. For all embryonic stages RIA occurred during comparable periods after irradiation, but in older animals its maximum shifted towards earlier post-irradiation time points. In summary, our results are in good agreement with data from the developing rodent retina, strengthening the suitability of the chick embryo for the analysis of the radiation response in the developing central nervous system.

## Introduction

The transfer of unrepaired DNA damage into prospective daughter cells by DNA duplication during S-phase or mitosis represents an important risk for the organism. Thus, accurate detection and repair of DNA damage occurring after genotoxic stress are preconditions for the maintenance of genomic integrity of dividing stem and progenitor cells during embryonic development. To minimize the risk of DNA damage propagation, cells must rely on cell cycle checkpoints, giving damaged cells ample time to repair the damage before they enter these critical cell cycle phases. Besides this, damaged cells might become eliminated via apoptosis when the degree of damage exceeds a specific threshold. In the developing central nervous system (CNS) of rodents, elimination of damaged cells by apoptosis is a major component of the response to ionizing radiation (IR) [1,2], whereas control of cell cycle progression through cell cycle checkpoints is limited. While recent studies confirmed the existence of a G2-arrest through the G2/M-checkpoint after DNA damage, no blockade of S-phase entry by a G1/S-checkpoint was detected [3,4].

The retina represents a comparably simple tissue of the CNS with only few cell types, whose spatio-temporal genesis during development is well characterized. Mitosis is primarily confined to the apical side, whereas proliferating cells exhibit interkinetic nuclear migration in the developing tissue, with DNA synthesis taking place at the inner (basal) margin [5]. Cell cycle exit and differentiation onset also shows a spatio-temporal dependency, as it has been shown at high spatial resolution for the chick retina [6,7]. The first cells that leave the cell cycle are located in the central part of the retina, from where differentiation spreads toward the peripheral regions near the lens. Thus, at late embryonic stages proliferation is higher in peripheral regions of the retina [8].

When exposed to ionizing radiation, the response of the retinal tissue is comparable to other parts of the brain: recently, distinct apoptotic waves were detected after irradiation of rodents, with proliferating and postmitotic cells dying at different times [9,10]. As differentiation of the tissue proceeds, the impact of IR on survival of retinal cells decreases [11].

By comparing the radiation response in the CNS of chick embryos with previous data from rodents, this study aimed to establish the chick embryo as a new animal model for the analysis of radiation effects on neural development. The chick not only represents a lower vertebrate, but also offers some further advantages over mammals. Most importantly, the mother has not to be sacrificed in order to gain access to the embryos. Additionally, the possibility to "window" fertilized eggs allows the determination of an embryonic stage before the experiment and makes the embryo highly accessible to experimental manipulations. Thus, the establishment of the chick embryo as a model system in the research of radiation effects on development allows us to address the National Institute of Health 3R policy to Reduce, Refine and Replace animals in research.

Here we present a detailed analysis of the radiation-induced activation and maintenance of cell cycle arrests via checkpoints and the time-, dose- and region-dependent occurrence of apoptotic events after irradiation in three selected developmental stages of the chick retina. Our data show the lack of a radiation-induced G1/S checkpoint in retinal progenitor cells, allowing cells to replicate their damaged DNA. In contrast, a rapid G2/M checkpoint activation efficiently inhibited the progression of irradiated cells into mitosis. Noticeably, cells that were released from this G2/M arrest still harbored unrepaired DNA damage. IR further induced apoptosis, whereby the dose-dependency as well as the temporal and spatial occurrence of apoptotic events drastically changed during development. In summary, our data are in good agreement with previous results obtained from rodent retina. Thus the chick embryo represents a suitable lower vertebrate model system for the research of the radiation response of the developing CNS.

## Materials and Methods

### *In ovo* irradiation of animals and tissue isolation

Eggs from the white leghorn chick (Gallus gallus domesticus) were incubated at 37°C and 65% humidity for 2–3 days. Then, a window was cut into the egg shell for Hamburger Hamilton (HH) stage determination and—if necessary—5-bromo-2'-deoxyuridine (BrdU) and 5-ethynyl-2’-deoxyuridine (EdU) application. Before treatment, HH stages were determined; the following stages were used: E3 HH 16–18, E5 HH 26–28, E7 HH 30–32. Irradiation was performed with 135 kV and 19 mA, using a Philips MCN 165/796704 X-Ray machine, equipped with a Tungsten anode and a Beryllium window. Doses of 0.5 Gy, 1 Gy, 2 Gy and 4 Gy were estimated by using an ion chamber (PTW) that was placed on top of the egg; the dose rate was 0.5 Gy / min. Dependent on the experiment, chick embryos were sacrificed by decapitation at 30 min, 1, 3, 6, 12, 24, 48 or 72 hours (hrs) after the treatment. For tissue isolation, eyes were collected in F-12 medium (Gibco) on ice. For immunohistochemistry, eyes were fixed in PBS containing 4% formalin (pH 7.3) for 16 hrs. Formalin-fixed tissues were embedded in paraffin and sectioned at a thickness of 3 μm.

Animal experiments were performed in strict accordance with German Animal Welfare legislation and were approved by the animal welfare representative of the TU Darmstadt (Tierschutzbeauftragte) and the Regierungspräsidium Darmstadt (Darmstadt, Germany).

### Immunofluorescence analysis of tissues

After dewaxing in xylene and rehydration, sections were incubated in citrate buffer for 1 h at 95°C. Sections were encircled with a liquid blocker (Pap Pen, Kisker Biotech) and incubated with primary antibodies for 5 hrs at 37°C or overnight at room temperature. Antibodies used were: BrdU from mouse (G3G4, DSHB) 1:500, Cleaved Caspase-3 from rabbit ((Asp 175) 5A1E Rabbit mAb #9664 Cell Signaling Technology Inc.) 1:1000, PCNA from mouse (PC10, #M8079 DAKO, Germany) 1:200, phospho-Histone H2A.X from mouse (Ser 139) clone JBW301 (05–636, Merck Millipore) 1:500, phospho Histone H3 (Ser-28) from goat (sc-12927, Santa Cruz Biotechnology Inc.) 1:200, Rad51 (Ab-1) from rabbit (PC130, Calbiochem/Merck Millipore, Lot# D00024394) 1:500, Visinin from mouse (7G4, DSHB) 1:500. Primary antibodies were diluted in blocking solution (5% BSA, 0,1% Triton X-100 in PBS) following Alexa488 or Alexa594-conjugated secondary antibodies (all made in donkey, 1:200, Dianova) for 2.5 hrs at room temperature and staining of DNA with DAPI.

### EdU and BrdU incorporation studies

In order to analyze radiation-induced G1/S checkpoint activation, BrdU and EdU incorporation studies were performed. For BrdU studies alone, 100 μl of a 25 mM BrdU solution (Boehringer, solved in PBS) were pipetted on the top of the embryo at various time points after irradiation. To check S-phase entry of irradiated cells at early times after irradiatin, BrdU incorporation was allowed to take place from 3 to 6 hrs after irradiation. To check for a late activation of the G1/S checkpoint, BrdU exposure was started 6 hrs after irradiation and lasted till 12 hrs post irradiation. Fixation was done as described above. For EdU-BrdU double labeling, 250 μl of a 500 μM EdU solution (Panatecs, solved in PBS) was added directly after irradiation, followed by the administration of 100 μl of 25 mM BrdU (solved in PBS) at 3 hrs post irradiation.

### FACS analysis of cell cycle distribution

6 hrs after irradiation of E5 and E7 embryos with 2 Gy, eyes were collected in F-12 medium (Gibco) on ice. Retinae were isolated in Hank’s balanced salt solution (Worthington Biochemicals) and dissociated by accutase (pAA) digestion for 30–45 minutes at 37°C. Digestion was stopped by adding DMEM incl. 10% FCS, followed by mechanical dissociation with a fire polished Pasteur pipette. Preceding FACS analysis, single cells were fixed in 70% ice cold ethanol, stained with propidium iodide (50 μg / ml in PBS) and incubated with 0.5 mg / ml RNase A (Epicentre) for 30 min in PBS. FACS analysis was performed on a FC500 (Beckman Coulter).

### Fluorescence Microscopy and image analysis

Images of retinae were taken on a confocal microscope (Leica TCS SP5 II) with LAS AF Lite software (Leica). Foci determination of the phosphorylated form of the H2AX histone (γ H2AX), phospho-Histone H3 positive mitotic cells, pyknotic nuclei and cleaved caspase 3 positive cells was performed on captured images. Localization studies of pyknotic nuclei were carried out in a standard sector of the retina next to the optic nerve head. Sectors of comparable thickness were divided into 5 equally sized bins in its horizontal dimension. The sector was aligned such that the first bin was at the presumptive photoreceptor layer of the retina, with its long axis parallel to the apical border of the tissue. The images were arranged using ImageJ and GraphPad Prism. All images are arranged such that the apical part of the retina (referred to the presumptive outer nuclear layer (pONL)) faces up- and the basal part downwards.

### Statistics

Statistics were performed using GraphPad Prism statistical software (version 6.07). If not indicated otherwise, at least three independent experiments were carried out. Unpaired two-tailed student’s *t*-test was used for testing of γ H2AX foci and BrdU quantifications after *in ovo* irradiations. One-way ANOVA with Tukey-test was used for significance testing of pH3-positive cell determination and the quantification and localization analysis of pyknotic nuclei. For all analyses a 95% confidence interval with *P < 0.05, **P < 0.01 and ***P < 0.001 was defined. The number of each experiment is indicated in the figure legends.

## Results

### Radiation induces the formation of γ H2AX and Rad51 foci in retinal progenitor cells

**Fig 1:**
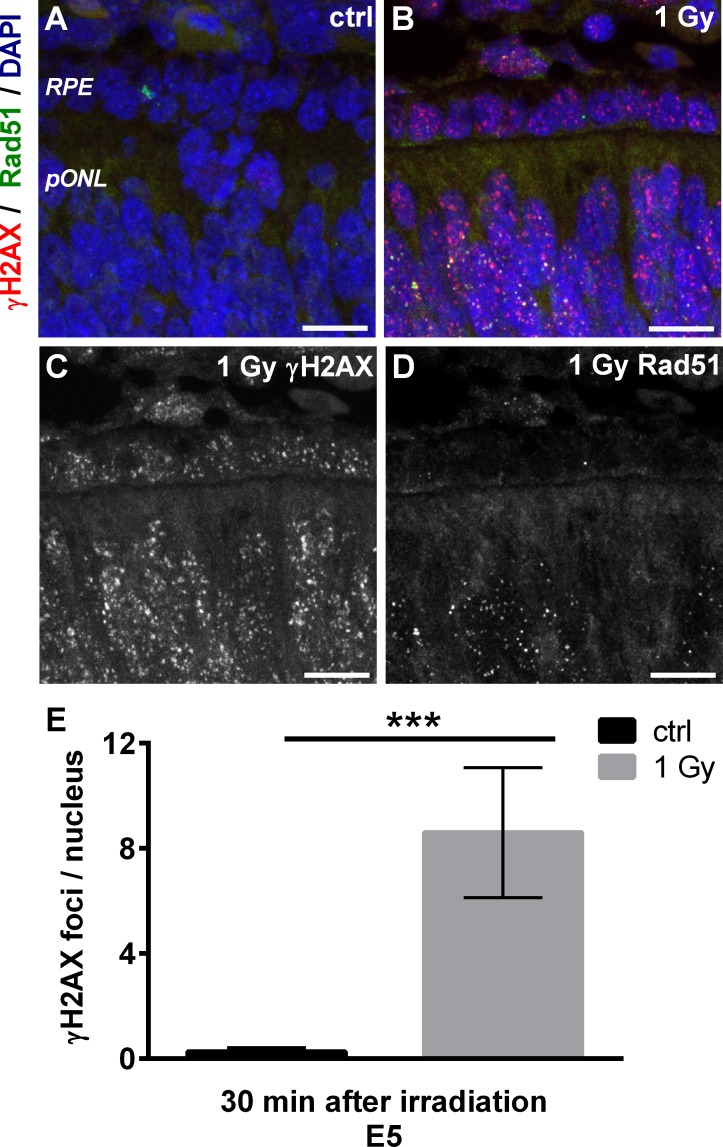
Cell type specific accumulation of γ H2AX and Rad51 foci at radiation induced DSBs. (A) γ H2AX (red) and Rad51 (green) staining in E5 control retina. Nuclei were counterstained with DAPI (blue). Note the absence of any signals. (B-D) γ H2AX (red) and Rad51 (green) staining in E5 retina at 30 min after irradiation. Nuclei were counterstained with DAPI (blue). Note that γ H2AX foci appear in all nuclei whereas Rad51 foci are only visible in some nuclei of the retina and the RPE. (E) Quantification of γ H2AX foci in RPE cells devoid of Rad51 foci at 30 min after irradiation with 1 Gy (black, non-irradiated control). Data are presented as means (n = 3, with 10 nuclei analyzed for each experiment) ± SEM. (*** P<0.001). Scale bar = 10 μm. RPE, retinal pigmented epithelium; pONL, presumptive outer nuclear layer.

Previous studies have shown a direct correlation between the radiation dose and the number of induced DNA double strand breaks (DSBs) in irradiated cells [12]. To confirm the correctness of our dosimetry we used the γH2AX foci assay to quantify DSBs in cells of the irradiated embryos. Staining against this general DSB marker show that E5 control retinae were devoid of γH2AX foci (Fig 1A), whereas at 30 min after irradiation an accumulation of distinct γ H2AX foci in all cells of the retina and the adjacent retinal pigmented epithelium (RPE) was observed (Fig 1B and 1C). Since the number of DSBs in a cell depends on its position in the cell cycle, leading to unintentional variance, we colabeled γ H2AX with Rad51, a protein which colocalizes to γ H2AX foci in S- and early G2-phase cells [13]. Thus, the colabeling allowed us to distinguish between cells in different phases of the cell cycle. In contrast to γ H2AX, much fewer cells exhibited Rad51 foci (Fig 1B and 1D). Double labeling of Rad51 with Visinin as a marker for photoreceptor precursors [14] showed no Rad51 foci in this postmitotic cell population (S1A Fig). In contrast, double labeling of Rad51 with PCNA and BrdU as markers for proliferating progenitors clearly showed an accumulation of Rad51 foci in those cells (S1B and S1C Fig). With the next step γ H2AX foci were counted in RPE cells which lacked S/G2-phase specific Rad51 foci. Cells with extraordinary high foci numbers were considered as late G2-phase cells and excluded from the data set. Quantification revealed 8 DSBs per nucleus 30 min after irradiation with 1 Gy (Fig 1E).

### No radiation-induced G1/S checkpoint but robust G2/M checkpoint activation in retinal progenitors

**Fig 2:**
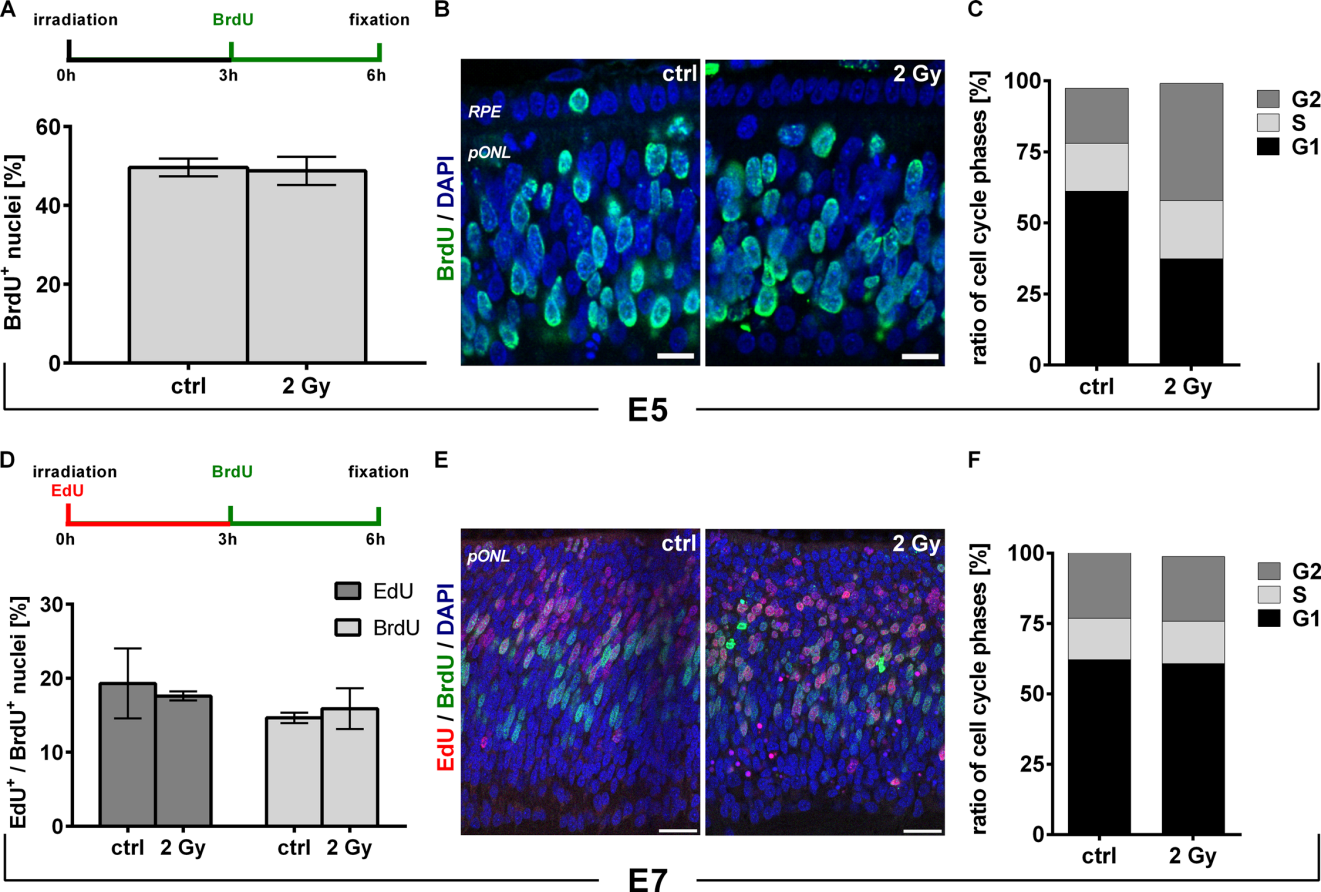
Lack of a G1/S checkpoint in retinal progenitor cells after DNA damage. (A) Scheme of experimental design and quantification of BrdU+ cells in E5 embryos. BrdU was added at 3 hrs after 2 Gy irradiation. Fixation was done at 6 hrs after irradiation. (B) Staining against BrdU (green) in retinae of E5 controls and embryos irradiated with 2 Gy. Nuclei were counterstained with DAPI (blue). (C) Quantification of FACS cell cycle analysis from retinae of control and 2 Gy irradiated E5 embryos at 6 hrs after irradiation (n = 2). No differences in the ratio of S-phase cells were observed. (D) Scheme of experimental design and quantification of EdU+ and BrdU+ cells in E7 embryos. EdU was added directly after 2 Gy irradiation and BrdU was additionally applied at 3 hrs. Fixation was done at 6 hrs after irradiation. (E) Staining against EdU (red) and BrdU (green) in retinae of E7 controls and embryos irradiated with 2 Gy. Nuclei were counterstained with DAPI (blue). (F) Quantification of FACS cell cycle analysis of retinae from control and 2 Gy irradiated E7 embryos at 6 hrs after the treatment (n = 4). No differences in the ratio of S-phase cells were observed. Data are presented as means (n = 3, with sectors analyzed in central retinal regions that contain at least 400 cells for each experiment) ± SEM. Scale bar = 10 μm (B) and 20μm (E). RPE, retinal pigmented epithelium; pONL, presumptive outer nuclear layer.

Recent studies have shown the existence of a radiation-induced G2-arrest by the G2/M-checkpoint but no blockade of S-phase entry by a G1/S-checkpoint in the developing CNS of mice [3,4]. To analyze the impact of radiation on S-phase entry in the chick embryo, we first performed BrdU labeling studies. Since the G1/S-checkpoint has been described to be slowly activated at 4–6 hrs after irradiation of primary fibroblasts *in vitro* [15], BrdU was administered to E5 embryos at 3 hrs after the treatment. Fixation was performed at 6 hrs after irradiation (Fig 2A). Quantifications of BrdU-positive cells (BrdU+) in central parts of the retina revealed no significant changes (p-value = 0.844) between controls (49%) and 2 Gy irradiated samples (48%). (Fig 2A and 2B). FACS analysis of cell cycle distribution at 6 hrs after the irradiation confirmed the lack of changes in S-phase population but showed an increase in G2 phase cells after 2 Gy (Fig 2C, see S2A Fig for FACS blots).

**Fig 3:**
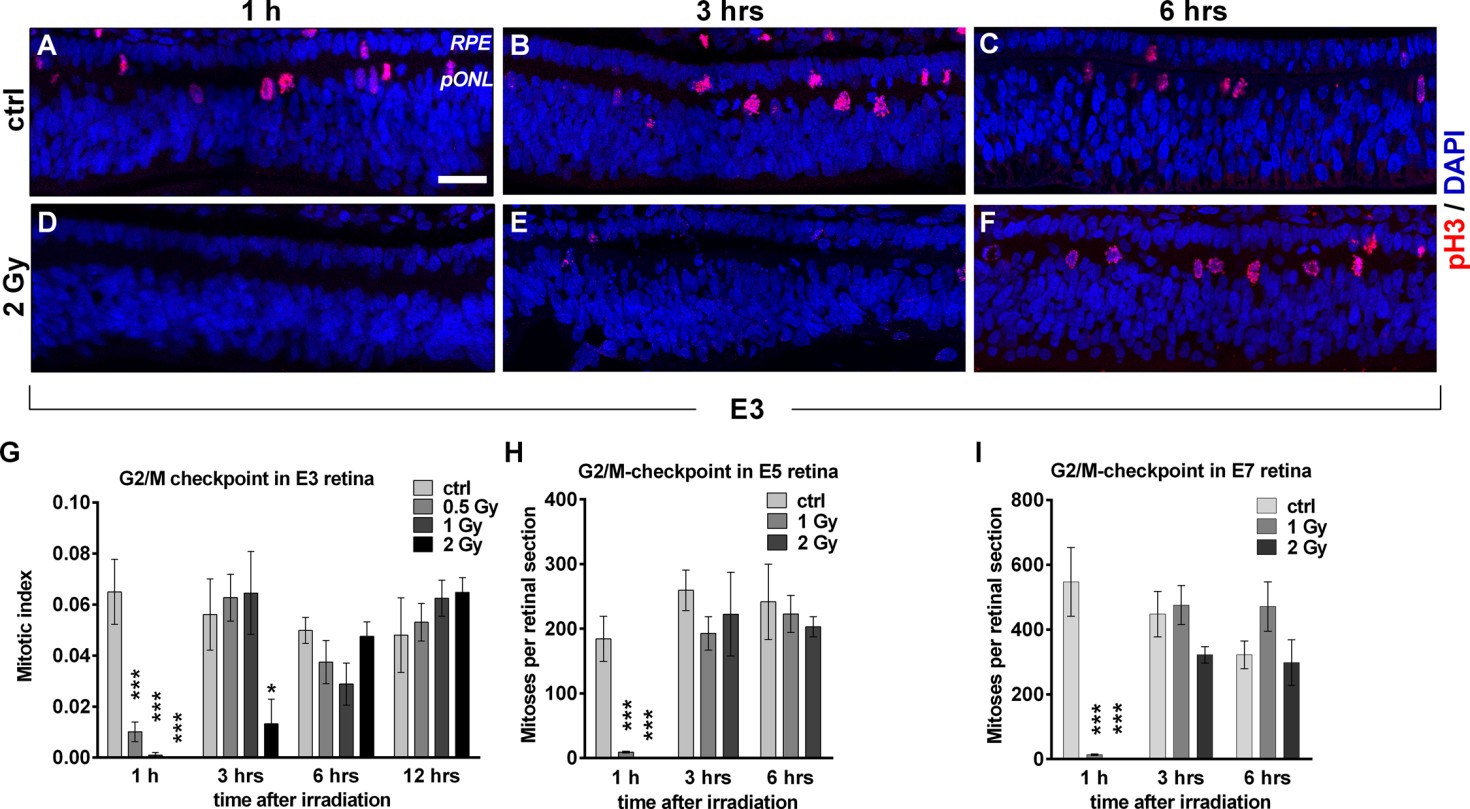
X-ray-induced cell cycle arrest at G2/M-checkpoint in embryonic chick retina is abrogated at about 3 hours post-irradiation throughout development. (A-F) Staining against the mitotic marker pH3 (red) in control (A-C) and 2 Gy (D-F) irradiated E3 retinae at 1, 3 and 6 hrs after irradiation. Nuclei were counterstained with DAPI (blue). Note absence of mitotic cells up to 3 hrs after irradiation. (G) Mitotic index in retinae of E3 embryos after irradiation with various doses at different time points. (H) Absolute numbers of mitotic events in retinal slices of E5 after irradiation with various doses at different time points. (I) Absolute numbers of mitotic events in retinal slices of E7 after irradiation with various doses at different time points. Note that at all stages, cells are released from G2-arrest around 3 hrs after irradiation. Data are presented as means (n = 3, with at least 100 cell analyzed for E3 and at least three different retinal slices analyzed for E5 and E7) ± SEM. (*P<0.05 **P< 0.01 *** P<0.001). Scale bar = 25 μm. RPE, retinal pigmented epithelium; pONL, presumptive outer nuclear layer.

To rule out earlier changes in S-phase entry, E7 embryos were double-labeled with EdU and BrdU. Hereby, EdU was administered directly after irradiation and BrdU was added in excess at 3 hrs. Fixation was done at 6 hrs after irradition (Fig 3D). No significant changes between both EdU (p-value = 0.739) and BrdU incorporation (p-value = 0.68) was detected between controls (17% for EdU, 13% for BrdU) and 2 Gy irradiated samples (16% for EdU and 10% for BrdU) (Fig 2D and 2E). FACS analysis at 6 hrs after the irradiation also showed no radiation-induced changes in cell cycle distribution (Fig 2F, see S2B Fig for FACS blots).

To check for later radiation-induced changes in S-phase entry, BrdU was applied to E5 and E7 embryos from 6 to 12 hrs after irradiation. Again, irradiation had no impact on the ratio of BrdU+ cells (S3 Fig).

In a final step we analyzed the radiation-induced activation of the G2/M checkpoint by staining against the phosphorylated histone H3 (pH3), allowing us to determine the numbers of mitotic events in untreated and irradiated retinae. Fig 3 shows a typical staining pattern of pH3 in an E3 control retina with mitotic cells located at the apical side of the tissue (Fig 3A–3C). 1 h after 2 Gy irradiation, pH3 positive cells were completely absent. 3 hrs after irradiation mitotic events were still reduced, but had reached again control levels at 6 hrs after irradiation (Fig 3D–3F). Quantification of mitotic events in E3 embryos revealed a dose-dependency of this G2/M-arrest (Fig 3G). Whereas nearly no more mitotic events were observed at 1 h after 0.5–2 Gy, at 3 hrs after irradiation only a dose of 2 Gy (p-value 0.039), but not 0.5–1 Gy, still had a significantly decreasing effect. However, 6 and 12 hrs after irradiation none of the analyzed doses presented any differences to controls. In E5 and E7 retinae, 1 and 2 Gy also completely diminished mitosis at 1 h after irradiation (Fig 3H–3I). At 3 hrs, numbers of mitotic events had returned to control levels, even after 2 Gy (see also S4 Fig).

To check whether G2/M checkpoint released retinal progenitor cells still harbour unrepaired DSBs, we analyzed those cells by staining against γ H2AX. At 30 min after irradiation with 2 Gy, residual mitotic cells showed massive γ H2AX signals (S5 Fig). 3 hrs after treatment the reoccurring mitotic cells still showed robust γ H2AX signals, albeit weaker than after 30 min.

### Decreasing sensitivity and changing temporal occurrence of radiation-induced apoptosis during development

**Fig 4:**
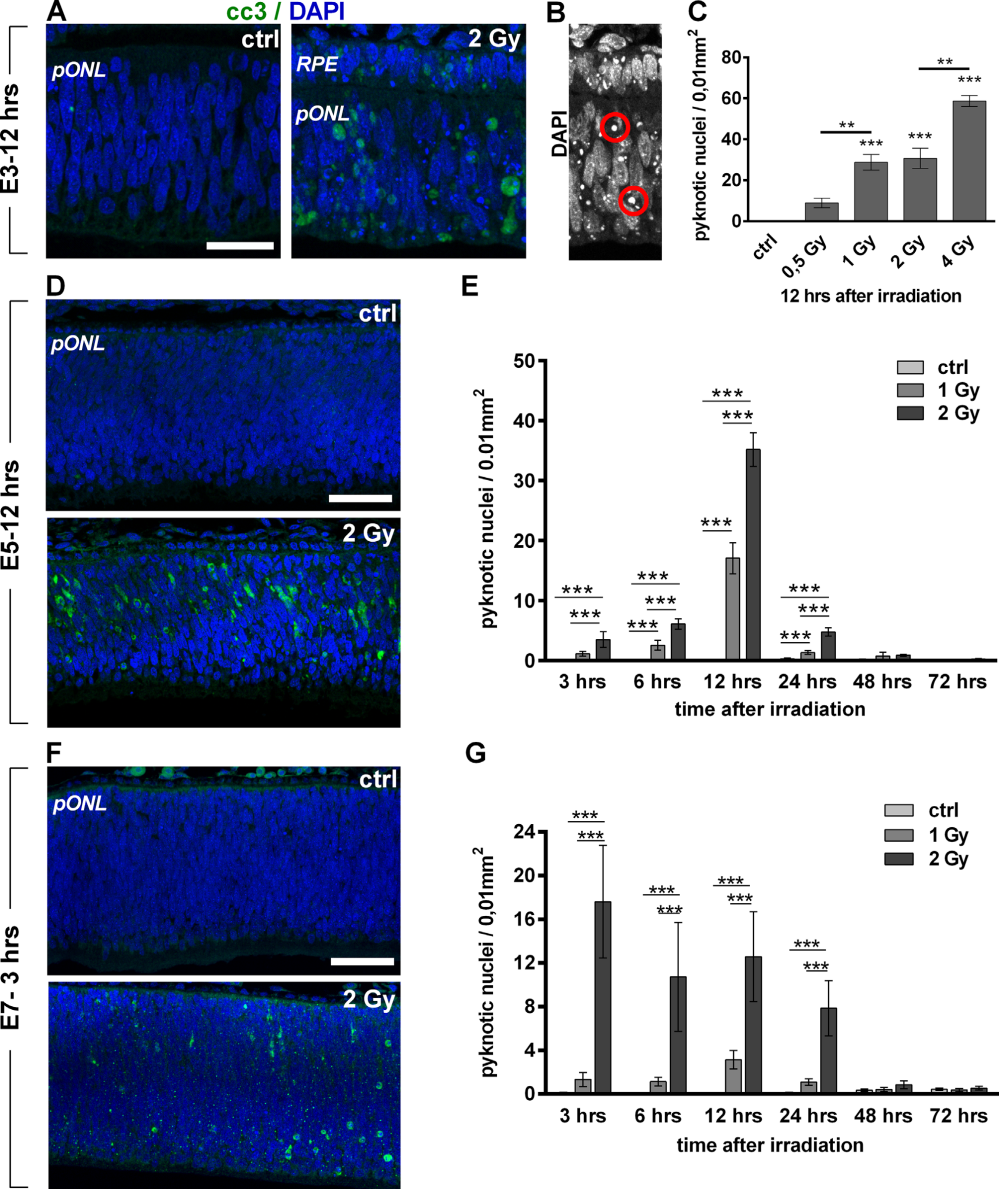
Radiation-induced apoptotic events occur time- and dose-dependently between different stages of retinal development. (A) cc3 staining (green) in control and in 2 Gy-irradiated E3 retinae at 12 hrs after irradiation. Nuclei were counterstained with DAPI (blue). (B) Representative DAPI staining of irradiated E3 retina including pyknotic nuclei (encircled in red) used for quantification of pyknosis. (C) Quantification of pyknotic nuclei at 12 hrs after irradiation with 0.5, 1, 2 and 4 Gy in central parts of E3 retinae. Note strong increase of apoptotic events after dose doubling from 0.5 to 1, but no further increase after doubling the dose from 1–2 Gy. (D) cc3 staining (green) in control and 2 Gy-irradiated E5 retinae at 12 hrs after irradiation. Nuclei were counterstained with DAPI (blue). (E) Quantification of pyknotic nuclei at 3–72 hrs after irradiation with 1 and 2 Gy in central parts of E5 retinae. Note the peak of radiation-induced apoptosis (RIA) at 12 hrs after irradiation and the linear correlation between doses and RIA. (F) cc3 staining (green) in control and 2 Gy-irradiated E7 retinae at 3 hrs after treatment. Nuclei were counterstained with DAPI (blue). (G) Quantification of pyknotic nuclei at 3–72 hrs after irradiation with 1 and 2 Gy in central parts of E7 retinae. Note the peak in RIA at 3 hrs after irradiation and absence of RIA after 1 Gy. Data are presented as means (n = 3 for E3, n = 7 for E5 and E7 with at least four different pictures analyzed for each experiment;) ± SEM (*P<0.05 **P< 0.01 *** P<0.001). Scale bar = 25 μm (A) and 50 μm (D, F). RPE, retinal pigmented epithelium; pONL, presumptive outer nuclear layer.

Radiation has been described to induce apoptotic waves in progenitor cells of the developing CNS (4;9;10). In order to characterize the temporal occurrence and the dose dependency of the apoptotic response in different developmental stages of the retina, this tissue was analyzed for the occurrence of dying cells at various time points after treatment. Hereby, pyknotic nuclei and cells positive for cleaved caspase 3 (cc3+) were used as established markers for RIA [4]. When analyzed 3, 6, 12 and 24 hrs after irradiation we observed a constant increase in the numbers of RIA in E3 retinae up to 12 hrs after a dose of 2 Gy (S6 Fig). 24 hrs after the treatment no more cc3+ cells could be detected. In order to quantify the dose dependency of RIA at this embryonic stage, pyknotic nuclei were quantified at 12 hrs after irradiation. Hereby we observed no significant increase from controls after 0.5 Gy, but a significant elevation to 29 pyknoses / 0,01 mm^2^ with a dose of 1Gy (Fig 4A–4C). Similar numbers of pyknotic nuclei were found after 2 Gy (30 / 0,01 mm^2^). This result correlated well with the quantification of cells positive for cc3 (S5I and S5J Fig). A dose of 4 Gy again lead to significantly increased pyknosis when compared to 2 Gy (59 / 0,01 mm^2^,^,^ p-value = 0.0086).

In the next step we quantified the temporal course of RIA from 3 to 72 hrs after 1 and 2 Gy in the central retina of E5 embryos by counting of pyknotic nuclei. Hereby, we observed significantly increased cell death until 24 hrs after the treatment with a maximum of RIA at 12 hrs after irradiation with both doses (Fig 4D and 4E). 48 and 72 hrs later, no significant differences to control levels were found. Quantification of RIA also revealed that at the peak time of RIA (12 hrs), the number of dying cells was doubled from 17 to 35 / 0.01mm^2^ by increasing the dose from 1 to 2 Gy (Fig 4E). Counting of cc3+ cells at the different time points revealed comparable data (S7 Fig). When exposed to 4 Gy, all of the E5 embryos (100%) died within 6 hrs, probably due to hemorrhages (n = 5, retinae were not analyzed). A more detailed analysis of RIA at the peak of RIA (12 hrs) revealed comparable numbers of dying cells in central and more peripheral regions, whereas the joint region (hinge) between the retina and RPE close to the lens were devoid of RIA (S7 Fig).

E7 retinae presented further changes in RIA. At this stage, quantifications of pyknotic nuclei revealed no significant increase of RIA in central retinae after 1 Gy from 3 to 72 hrs after the treatment (Fig 4G). In contrast, irradiation with 2 Gy was sufficient to induce RIA. Remarkably, cellular death occurred in a completely different time course when compared to E3 and E5 retina. Now, apoptosis peaked 3 hrs after irradiation with 17 apoptotic events per 0.01 mm^2^ and remained between 9 and 13 events until 24 hrs after the treatment. Determination of cc3 activity in lysates of whole retinae of E7 embryos after *in ovo* irradiation confirmed these results (S8 Fig). No RIA was detected at 48 and 72 hrs after this dose (Fig 4G). Further analysis of RIA at its peak time (3 hrs) revealed more dying cells in peripheral than in central parts of the retina after 2 Gy, but not after 1 Gy (S7 Fig). Hinge regions were again devoid of RIA even after 2 Gy (S8 Fig).

### Temporal differences in the cell death of differentiated and still proliferating cells

**Fig 5:**
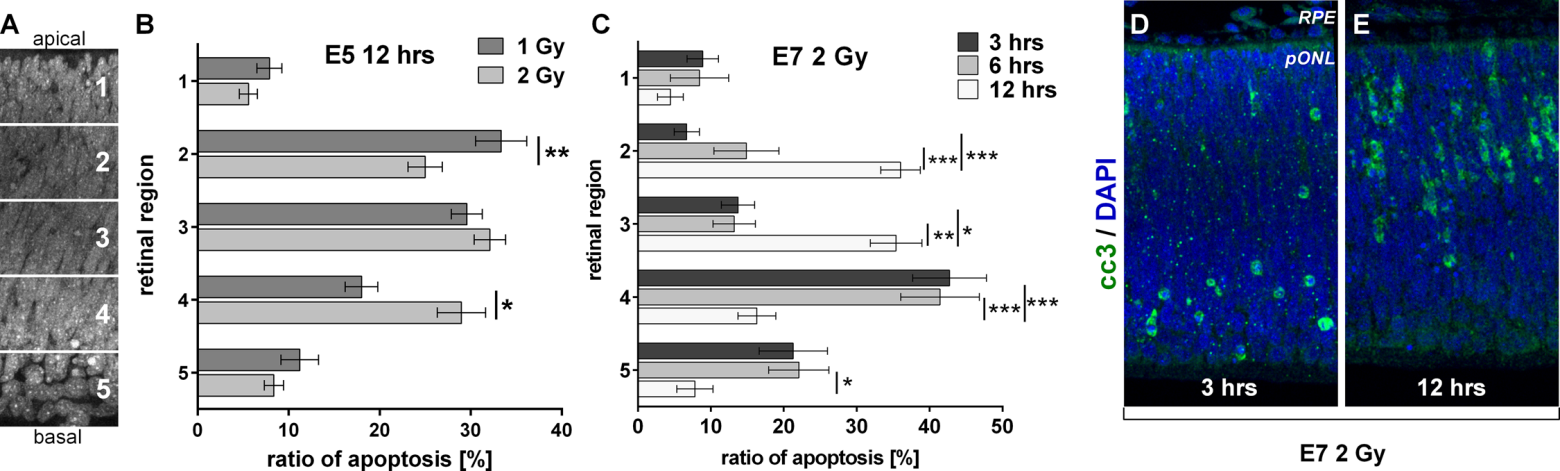
Radiation-induced apoptotic events depend on basal-apical cell positions in E7 retina. (A) For determination of the apical basal-to-apical positions of pyknotic nuclei within the retina, the tissue was subdivided in 5 bins ranging from the apical (No. 1) to the basal side (No. 5). (B) Quantification of pyknotic nuclei in E5 retinae 12 hrs after irradiation with 1 and 2 Gy. Note a slight shift in localization from apical to basal after doubling the dose from 1 to 2 Gy at 12 hrs. (C) Quantification of pyknotic nuclei in E7 retinae 3, 6 and 12 hrs after irradiation with 2 Gy. (D, E) Staining against cc3 (green) in E7 retinae at 3 and 12 hrs after irradiation with 2 Gy; nuclei were stained with DAPI (blue). Note a shift of apoptotic cells from basal to apical parts of the tissue from 6 to 12 hrs. Data are presented as means (n = 3, with at least four different pictures analyzed for each experiment); ± SEM (*P<0.05 **P< 0.01 *** P<0.001).

Noticeably, the dose dependency of RIA in the chick retina varied between the different embyronic stages that were analyzed in this study. In E5 retina, a dose doubling led to consistent doubling of RIA at all time points analyzed (Fig 4C). To gain more information about the cells that were additionally affected after dose doubling, we determined the spatial position of the dying cells in the retina. Since the spatial distribution of still proliferating and already postmitotic cells is well characterized for the retina, a determination of the position of dying cells indicates their actual differentiative state. To this end, retinae were subdivided into 5 bins from the apical (bin 1) to the basal side (bin 5) of the tissue (Fig 5A). Then, the ratio of apoptotic nuclei in each of these bins was counted. As shown in Fig 5B the increase of the dose from 1 to 2 Gy resulted in a significant increase of apoptotic cells from 18 to 29% (p-value 0.0019) in the basal part of the tissue (bin 4). We next determined the distribution of apoptotic nuclei in E7 retina at 3, 6, and 12 hrs after irradiation with 2 Gy. At 3 and 6 hrs after treatment, more than 60% of RIA were detected in the basal part of the retina (bin 4 and 5) (Fig 5C and 5D). In contrast, only 25% of the dying cells were located in these bins after 12 hrs. whereas nearly 70% localized to bin 2 and 3 (Fig 5C and 5E).

## Discussion

Here we analyzed responses to radiation at various doses in the chicken retina at different embryonic stages of development. Similar to the developing retina of mice [16], irradiation induced the formation of γ H2AX foci as a general marker for DSB repair in all retinal cells and the adjacent RPE of the chick embryo. The restriction of Rad51 foci to still proliferating cells is also in agreement with previous studies that refer to this protein as a major player in homologous recombination which is restricted to S- and early G2-phase cells in human cell lines and even the chicken B cell line DT40 [17]. Based on these findings, we assume that our γ H2AX foci quantification in cells devoid of Rad51 foci was restricted to G1 and / or G0 cells. Our results show similar numbers of γ H2AX foci in the chick retina (8 foci at 30 min after irradiation) when compared to mice (9 foci at 15 min after irradiation) [16]. Thus, irradiation with comparable doses leads to the formation of comparable numbers of γ H2AX foci in both developing chick and mouse retina.

With an established radiation setup we next aimed to elucidate the impact of ionizing radiation on cell cycle progression of proliferating retinal progenitors. The DNA damage-induced blockade of S-phase entry by the G1/S checkpoint has been described to depend on the transcriptional activation of several S-phase entry blocking genes, including p21 [18]. During this activation period only a slowing down of S-phase entry can be observed [19]. For the E6 chick retina, a p53-dependent increase in p21 mRNA levels after cisplatin-induced DNA damage has been reported [20]. Thus, it was surprising to see no effect on S-phase entry after radiation-induced DNA damage, neither by thymidine analogue incorporation studies nor FACS-based cell cycle distribution analysis. This missing effect might be explained by studies of the developing mouse cortex, which also showed a DNA damage-induced increase in p21 expression that was restricted to postmitotic cells where it triggered apoptosis. Proliferating cells instead showed nearly no increase in p21 expression, probably due to its repression by several transcription factors, which are responsible for the amplification of neuronal progenitor cells [21,22]. These transcription factors include Bmi1 and Olig2, which both can be found in the developing retina, indicating similar reasons for the lack of a G1/S-checkpoint in this tissue [23,24]. Thus, our results from the chick retina are in good agreement with data from the developing CNS of mice where no radiation-induced blockade of S-phase entry by a G1/S-checkpoint was detected [3,4].

The robust G2/M checkpoint activation by IR throughout development, as measured by mitotic indices, is another finding that is in accordance with data from the developing mouse cortex, although the duration of this mitosis blockage was shorter in chick than in mouse retinae [4]. For human fibroblasts, effective G2/M checkpoint activation needs a DSB level of 10–20 DSBs per nucleus [19]. Assuming a linear dose-damage relationship after *in vivo* irradiations [12,25], a dose of 0.5 Gy with our setup should induce about 5 DSBs per nucleus. Thus, G2/M checkpoint activation seems to be more efficient in retinal tissue when compared to fibroblasts, since at this dose mitotic events are nearly completely abolished after 1 h. In contrast, the dose-independent release from this checkpoint after 3 hrs with γ H2AX-marked DSBs still being present (see S5 Fig) confirms that the inherent insensitivity of the G2/M checkpoint maintenance which was described for human fibroblasts [15,19] is also existent in neuronal tissue.

Radiation-induced DNA damage is well known to induce apoptotic events in the developing CNS, whereby in the rodent retina the impact of IR on survival of retinal cells decreases as differentiation of the tissue proceeds [11]. According to this, we could not only show an ongoing desensitization of retinal tissue against RIA with progressing differentiation, but also the occurrence of a gradient in itself at later stages of development (see higher numbers of RIA in periperal than in central regions at E7 in S8 Fig). This latter gradient clearly corresponds to the earlier differentiation onset in the center of this tissue [7]. The low numbers of RIA in the hinge parts of the retina (see S7 and S8 Figs) throughout development are remarkable, since its progenitor cells are distinct from progenitors of the central and intermediate retina, regarding their molecular marker expression, proliferation behavior and their capacity to regenerate retinal tissue [8,26,27].

Besides the age-related decrease in radiosensitivity, the varying dose-dependency of RIA at specific embryonic stages was another finding of our studies. Recent studies have described a linear increase of RIA in the developing brain of E13.5 mice following its exposure from 10–200 mGy X-rays which directly correlated to the numbers of DSBs which were induced by the treatment [12]. In contrast, whole brain irradiation of adult mice induced apoptosis in the neural precursor cells from the subgranular zone of the hippocampal dentate gyrus whose extent was dose-dependent, but not in a linear way [28].

Comparing the ratio of RIA that was induced by irradiation with 1 and 2 Gy at three embryonic stages, E3 retina showed no further increase. In contrast, E5 RIA rose by a factor of 2 in E5, and a factor of 4 in E7 chicken embryos at 12 hrs after irradiation. With a 12-fold increase 3 hrs after irradiation in E7 retinae, this effect was even more striking at the time where RIA had its peak at this embryonic stage. These results indicate a change within the thresholds for apoptosis induction between the different embryonic stages. Such thresholds may be defined by a priming of the apoptotic machinery, e.g. the regulation of expression of pro- and anti-apoptotic proteins, as described for differentiating neuronal cells as well as pluripotent stem cells [29,30]. Our results now showed an increase in RIA after dose doubling at the basal side of the E5 retina. Since postmitotic cells starts to amass in this region of the tissue at this developmental stage, we assume that these cells have a higher apoptosis threshold than still proliferating cells.

The shift in the temporal occurrence of RIA to earlier post-irradiation time points, which was pronounced between E5 and E7 retina, was another striking result of our studies. Recently, differences in the temporal occurrence of apoptosis have been described for a single developmental stage of mouse and rat retina, with postmitotic cells dying at earlier post-irradiation time points than still proliferating cells [9]. Consistently, in the E7 retina early dying cells were mostly located in the basal part of the tissue which corresponds to the non-proliferative zone where prospective ganglion and amacrine cells are located [31]. The fact that E7 retinae, which contain much higher numbers of postmitotic cells than E5 retinae show an earlier apoptotic response further supports the idea, that early dying cells in the chick retina belong to the postmitotic population. Later dying cells were–in turn–located on the apical side, which represents the proliferative zone [31]. Thus, later dying cells might belong to the population of cells that has been irradiated during S-phase. These cells might still undergo mitosis and die after re-entry into S-phase as it has been described for the rat retina [9]. Thus, the temporal course of RIA in proliferating and postmitotic cells of the chick is again highly comparable to mammals.

## Conclusion

In this study we could show that the chick embryo is an appropriate model organism to analyze developmentally regulated radiation responses of the developing CNS. Our results showed a lack of a radiation-induced G1/S and the presence of a G2/M checkpoint in the chicken embryonic retina. Furthermore, the chick shows—similar to rodents—a decreasing tissue sensitivity and striking differences in the temporal occurrence of RIA with the progression of retinal development. Early and late occurring apoptotic events were shown to belong to different, namely to postmitotic and still proliferating cell populations, respectively. In summary, these results are in good agreement with data of mammalian model organisms. Thus, the chick represents a lower vertebrate model system, whose usage in the research of radiation effects on the developing CNS might help to reduce, refine and replace animal experiments with mammalian organisms.

## Supporting Information

S1 Fig Radiation-induced Rad51 foci are restricted to proliferative cells. (A) Rad51 (red) and Visinin (green) staining of E7 retina at 1 h after 2 Gy irradiation. Nuclei were counterstained with DAPI (blue). (B) Rad51 (red) and PCNA (green) staining of E7 retina at 1 h after 2 Gy irradiation. Nuclei were counterstained with DAPI (blue). (C) Rad51 (red) and BrdU (green) staining of E7 retina at 1 h after 2 Gy irradiation. Nuclei were counterstained with DAPI (blue). Note that Rad51 foci are not present in postmitotic, Visinin-positive photoreceptor precursors but highly abundant in cells positive for the proliferation markers PCNA and BrdU. Scale bar = 5 μm.
(PDF)

S2 Fig FACS blots of controls and irradiated retinae of E5 and E7 embryos. (A) Represenative FACS blots of the cell cycle distribution in retinal cells of E5 controls and embryos at 6 hrs after irradiation with 2 Gy. (B). Representative FACS blot of the cell cycle distribution in retinal cells of E7 controls and embryos at 6 hrs after irradiation with 2 Gy.
(PDF)

S3 Fig Scheme of experimental design and quantification of BrdU+ cells in E5 embryos. BrdU was added at 6 hrs after 2 Gy irradiation. Fixation was done at 12 hrs after irradiation. (B) Staining against BrdU (green) in retinae of E5 controls and embryos irradiated with 2 Gy. Nuclei were counterstained with DAPI (blue). (C) Scheme of experimental design and quantification of BrdU+ cells in E7 embryos. BrdU was added at 6 hrs after 2 Gy irradiation. Fixation was done at 12 hrs after irradiation. (D) Staining against BrdU (green) in retinae of E7 controls and embryos irradiated with 2 Gy. Nuclei were counterstained with DAPI (blue). Data are presented as means (n = 3, with sectors analyzed in central retinal regions that contain at least 300 cells for each experiment) ± SEM. Scale bar = 10 μm. RPE, retinal pigmented epithelium; pONL, presumptive outer nuclear layer.
(PDF)

S4 Fig Radiation-induced G2/M checkpoint is abrogated at 3 hrs after irradiation in E5 and E7 retina. pH3 staining (red) in control and 2 Gy irradiated retinae of E5 and E7 embryos. Nuclei were counterstained with DAPI (blue). No differences in the amount of pH3 positive cells were detected, although irradiated E7 retinae revealed high amounts of pyknotic nuclei. Scale bar = 50 μm. RPE, retinal pigmented epithelium; pONL, presumptive outer nuclear layer.
(PDF)

S5 Fig Mitotic cells still harbor unrepaired DSBs after G2/M checkpoint release. (A) Representative γH2AX (red) staining of a mitotic cell in E7 retina at 30 min after 2 Gy irradiation. Nuclei were counterstained with DAPI (blue). (B) Representative γH2AX (red) staining of a mitotic cell in E7 retina at 3 hrs after 2 Gy irradiation. Nuclei were counterstained with DAPI (blue). Note that cells that enter mitosis 3 hrs after the treatment still harbor unrepaired DSBs. Scale bar = 2 μm.
(PDF)

S6 Fig Radiation induced apoptosis peaks at 12 hrs after irradiation in E3 retina. (A-H) cc3 staining (green) in control and 2 Gy irradiated E3 retinae at 3, 6, 12 and 24 hrs after treatment. Nuclei were counterstained with DAPI (blue). Note highest abundance of apoptotic cells at 12 hrs after treatment. (I-J) Quantification of cc3^+^ cells (green, encircled in K) 12 hrs after irradiation with 0.5, 1 and 2 Gy in central parts of E3 retinae. Note strong increase of apoptotic events after dose doubling from 0.5 to 1, but no further increase after doubling the dose from 1–2 Gy. Data are presented as means (n = 3, with at least four different pictures analyzed for each experiment) ± SEM (*P<0.05 **P< 0.01 *** P<0.001). Scale bar = 25 μm. RPE, retinal pigmented epithelium; pONL presumptive outer nuclear layer.
(PDF)

S7 Fig Radiation induced apoptosis peaks at 12 hrs after irradiation in E5 retina. (A-I) cc3 staining (green) in control, 1 and 2 Gy irradiated E5 retinae at 12 hrs after treatment. Nuclei were counterstained with DAPI (blue). Note absence of cc3^+^ cells in central retina, and also their strong increase from 1 to 2 Gy in intermediate and central parts of the retina. (J) Quantification of cc3+ cells in central E5 retina at defined time points after irradiation with 1 and 2 Gy. RIA peak 12 hrs after irradiation; note correlation between doses and RIA, and also between appearance of cc3+ and pyknotic nuclei. Data are presented as means (n = 2) ± SEM (*** P<0.001). Scale bar = 50 μm. RPE, retinal pigmented epithelium; pONL, presumptive outer nuclear layer.
(PDF)

S8 Fig Radiation induced apoptosis peaks at 3 hrs after irradiation in E7 retina. (A-I) cc3 staining (green) in control, 1 and 2 Gy irradiated E7 retinae at 3 hrs after treatment. Nuclei were counterstained with DAPI (blue). Note absence of RIA after 1 Gy, and—after 2 Gy (C, F, I)—higher numbers in peripheral than in central parts of the retina. (J) Determination of cc3 activity in lysates of whole E7 retinae from controls and embryos irradiated with 2 Gy at defined time points after treatment. Data are presented as means (n = 3) ± SEM (*** P<0.001). Scale bar = 50 μm. RPE, retinal pigmented epithelium; pONL, presumptive outer nuclear layer.
(PDF)

S9 Fig Supplemental Experimental Procedures. (PDF)
