# Supplementary material for: Cell Cycle Regulation and Apoptotic Responses of the Embryonic Chick Retina by Ionizing Radiation
Source: PLoS One. 2016 May 10;11(5):e0155093. doi: 10.1371/journal.pone.0155093 (PMC4862647; doi:10.1371/journal.pone.0155093)
Supplement: S1 Fig — (A) Rad51 (red) and Visinin (green) staining of E7 retina at 1 h after 2 Gy irradiation. Nuclei were counterstained with DAPI (blue). (B) Rad51 (red) and PCNA (green) staining of E7 retina at 1 h after 2 Gy irradiation. Nuclei were counterstained with DAPI (blue). (C) Rad51 (red) and BrdU (green) staining of E7 retina at 1 h after 2 Gy irradiation. Nuclei were counterstained with DAPI (blue). Note that Rad51 foci are not present in postmitotic, Visinin-positive photoreceptor precursors but highly abundant in cells positive for the proliferation markers PCNA and BrdU. Scale bar = 5 μm. (PDF) [file pone.0155093.s001.pdf]

Supplemental Figures

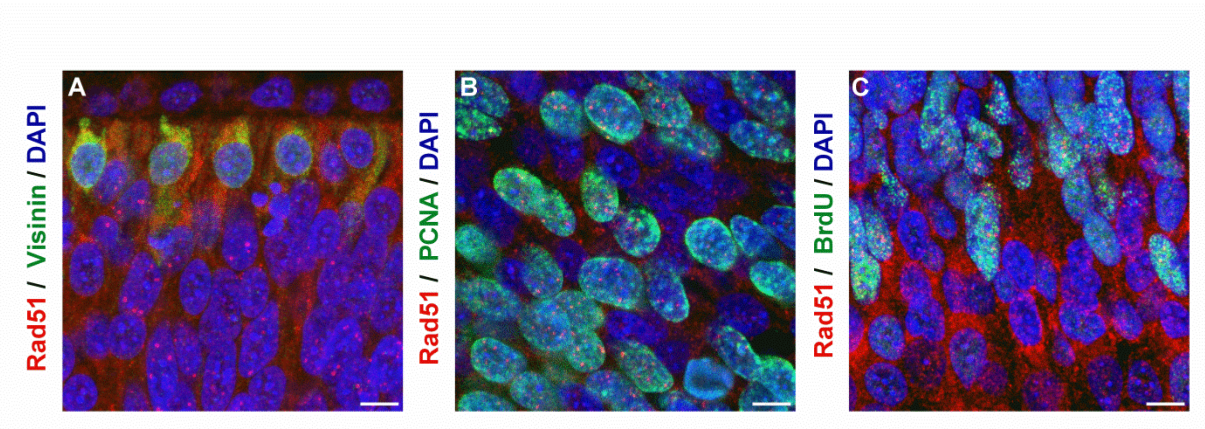

S1 Fig. Radiation-induced Rad51 foci are restricted to proliferative cells. (A) Rad51 (red) and Visinin (green) staining of E7 retina at 1 h after 2 Gy irradiation. Nuclei were counterstained with DAPI (blue). (B) Rad51 (red) and PCNA (green) staining of E7 retina at 1 h after 2 Gy irradiation. Nuclei were counterstained with DAPI (blue). (C) Rad51 (red) and BrdU (green) staining of E7 retina at 1 h after 2 Gy irradiation. Nuclei were counterstained with DAPI (blue). Note that Rad51 foci are not present in postmitotic, Visinin-positive photoreceptor precursors but highly abundant in cells positive for the proliferation markers PCNA and BrdU. Scale bar = 5 μm.
