# Supplementary material for: Cell Cycle Regulation and Apoptotic Responses of the Embryonic Chick Retina by Ionizing Radiation
Source: PLoS One. 2016 May 10;11(5):e0155093. doi: 10.1371/journal.pone.0155093 (PMC4862647; doi:10.1371/journal.pone.0155093)
Supplement: S2 Fig — (A) Represenative FACS blots of the cell cycle distribution in retinal cells of E5 controls and embryos at 6 hrs after irradiation with 2 Gy. (B). Representative FACS blot of the cell cycle distribution in retinal cells of E7 controls and embryos at 6 hrs after irradiation with 2 Gy. (PDF) [file pone.0155093.s002.pdf]

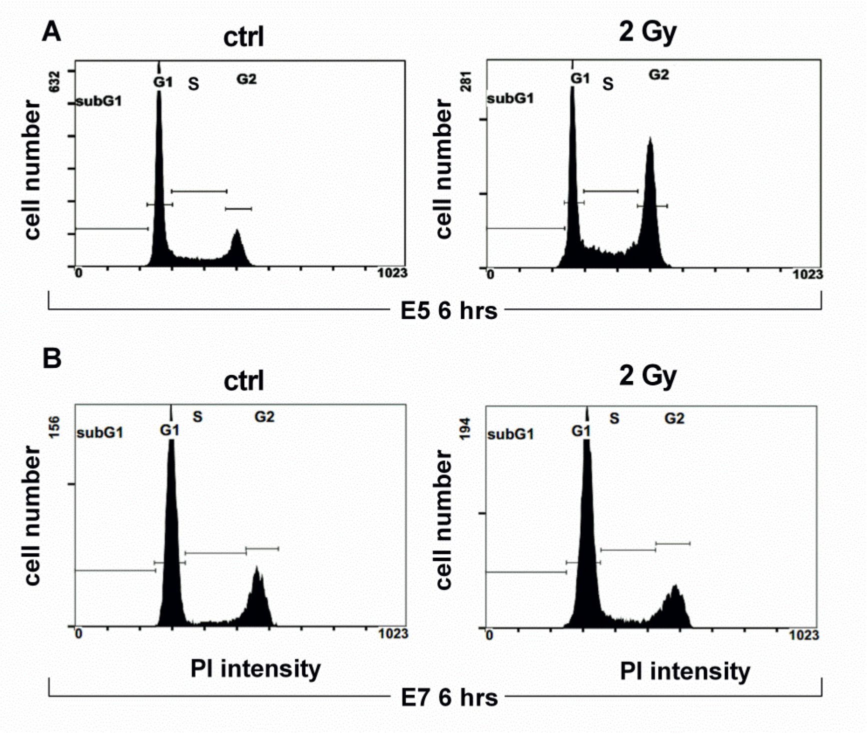

S2 Fig. FACS blots of controls and irradiated retinae of E5 and E7 embryos. (A) Represenative FACS blots of the cell cycle distribution in retinal cells of E5 controls and embryos at 6 hrs after irradiation with 2 Gy. (B). Representative FACS blot of the cell cycle distribution in retinal cells of E7 controls and embryos at 6 hrs after irradiation with 2 Gy.
