# Supplementary material for: Cell Cycle Regulation and Apoptotic Responses of the Embryonic Chick Retina by Ionizing Radiation
Source: PLoS One. 2016 May 10;11(5):e0155093. doi: 10.1371/journal.pone.0155093 (PMC4862647; doi:10.1371/journal.pone.0155093)
Supplement: S3 Fig — BrdU was added at 6 hrs after 2 Gy irradiation. Fixation was done at 12 hrs after irradiation. (B) Staining against BrdU (green) in retinae of E5 controls and embryos irradiated with 2 Gy. Nuclei were counterstained with DAPI (blue). (C) Scheme of experimental design and quantification of BrdU+ cells in E7 embryos. BrdU was added at 6 hrs after 2 Gy irradiation. Fixation was done at 12 hrs after irradiation. (D) Staining against BrdU (green) in retinae of E7 controls and embryos irradiated with 2 Gy. Nuclei were counterstained with DAPI (blue). Data are presented as means (n = 3, with sectors analyzed in central retinal regions that contain at least 300 cells for each experiment) ± SEM. Scale bar = 10 μm. RPE, retinal pigmented epithelium; pONL, presumptive outer nuclear layer. (PDF) [file pone.0155093.s003.pdf]

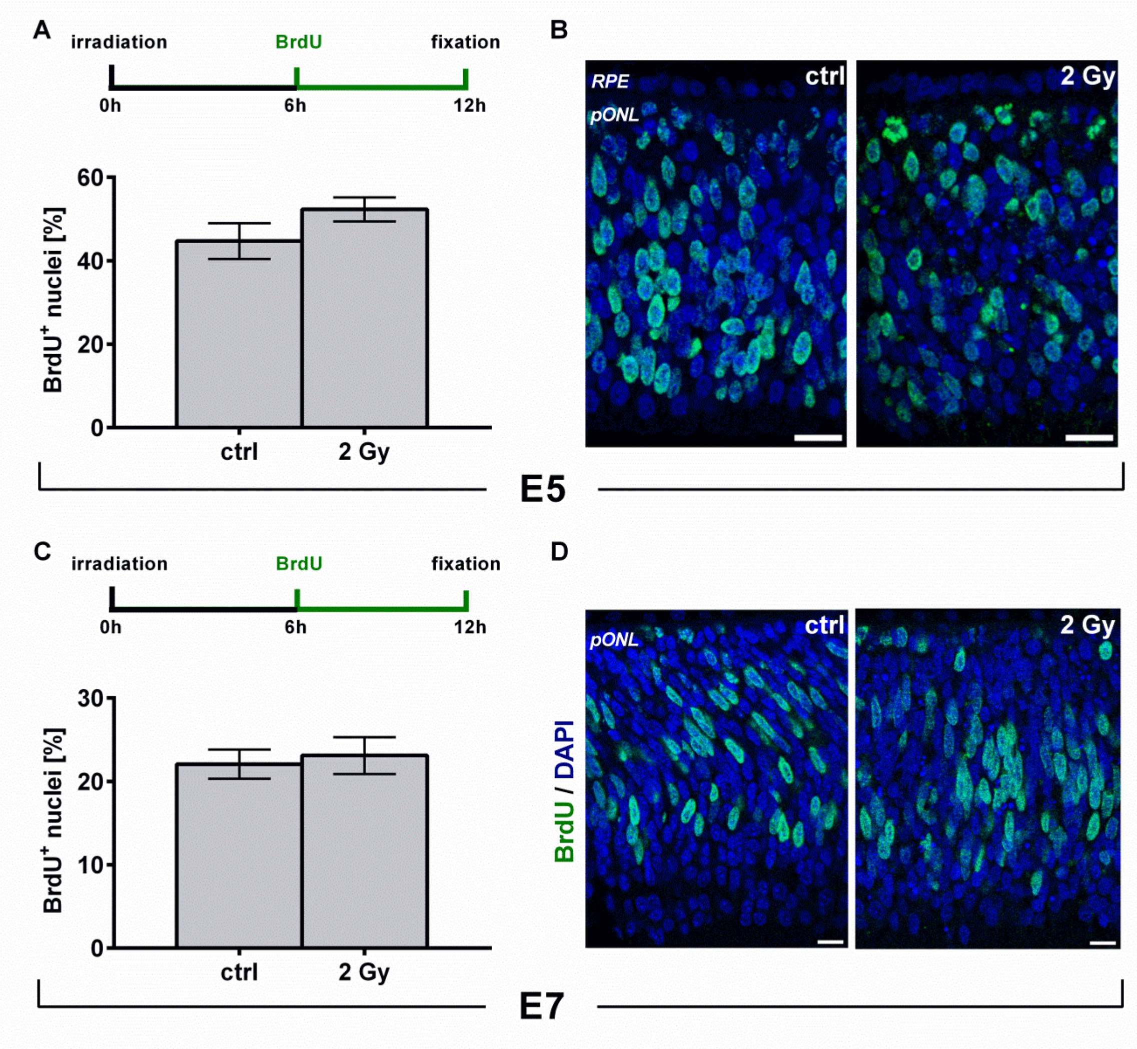

S3 Fig. Lack of a lately activated G1/S checkpoint in retinal progenitor cells after DNA damage. Scheme of experimental design and quantification of BrdU+ cells in E5 embryos. BrdU was added at 6 hrs after 2 Gy irradiation. Fixation was done at 12 hrs after irradiation. (B) Staining against BrdU (green) in retinae of E5 controls and embryos irradiated with 2 Gy. Nuclei were counterstained with DAPI (blue). (C) Scheme of experimental design and quantification of BrdU+ cells in E7 embryos. BrdU was added at 6 hrs after 2 Gy irradiation. Fixation was done at 12 hrs after irradiation. (D) Staining against BrdU (green) in retinae of E7 controls and embryos irradiated with 2 Gy. Nuclei were counterstained with DAPI (blue). Data are presented as means (n = 3, with sectors analyzed in central retinal regions that contain at least 300 cells for each experiment) ± SEM. Scale bar = 10 µm. RPE, retinal pigmented epithelium; pONL, presumptive outer nuclear layer.
