# Supplementary material for: Cell Cycle Regulation and Apoptotic Responses of the Embryonic Chick Retina by Ionizing Radiation
Source: PLoS One. 2016 May 10;11(5):e0155093. doi: 10.1371/journal.pone.0155093 (PMC4862647; doi:10.1371/journal.pone.0155093)
Supplement: S4 Fig — pH3 staining (red) in control and 2 Gy irradiated retinae of E5 and E7 embryos. Nuclei were counterstained with DAPI (blue). No differences in the amount of pH3 positive cells were detected, although irradiated E7 retinae revealed high amounts of pyknotic nuclei. Scale bar = 50 μm. RPE, retinal pigmented epithelium; pONL, presumptive outer nuclear layer. (PDF) [file pone.0155093.s004.pdf]

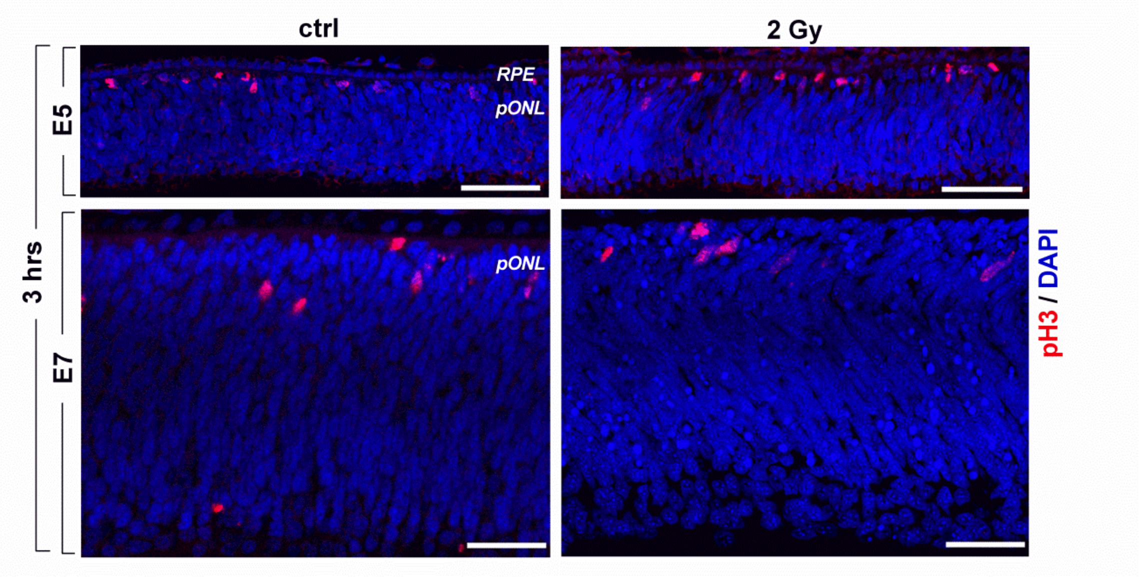

S4 Fig. Radiation-induced G2/M checkpoint is abrogated at 3 hrs after irradiation in E5 and E7 retina. pH3 staining (red) in control and 2 Gy irradiated retinae of E5 and E7 embryos. Nuclei were counterstained with DAPI (blue). No differences in the amount of pH3 positive cells were detected, although irradiated E7 retinae revealed high amounts of pyknotic nuclei. Scale bar = 50 μm. RPE, retinal pigmented epithelium; pONL, presumptive outer nuclear layer.
