# Supplementary material for: Cell Cycle Regulation and Apoptotic Responses of the Embryonic Chick Retina by Ionizing Radiation
Source: PLoS One. 2016 May 10;11(5):e0155093. doi: 10.1371/journal.pone.0155093 (PMC4862647; doi:10.1371/journal.pone.0155093)
Supplement: S5 Fig — (A) Representative γH2AX (red) staining of a mitotic cell in E7 retina at 30 min after 2 Gy irradiation. Nuclei were counterstained with DAPI (blue). (B) Representative γH2AX (red) staining of a mitotic cell in E7 retina at 3 hrs after 2 Gy irradiation. Nuclei were counterstained with DAPI (blue). Note that cells that enter mitosis 3 hrs after the treatment still harbor unrepaired DSBs. Scale bar = 2 μm. (PDF) [file pone.0155093.s005.pdf]

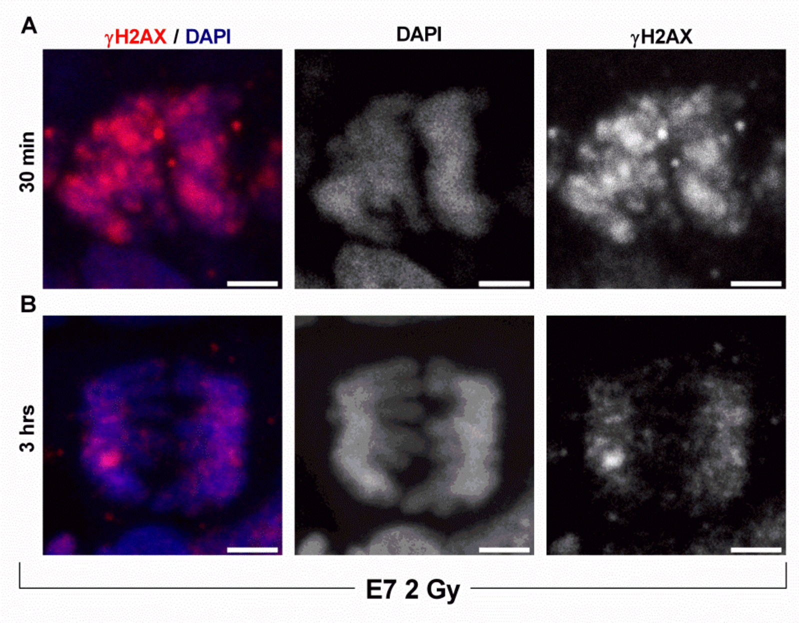

S5 Fig. Mitotic cells still harbor unrepaired DSBs after G2/M checkpoint release. (A) Representative γH2AX (red) staining of a mitotic cell in E7 retina at 30 min after 2 Gy irradiation. Nuclei were counterstained with DAPI (blue). (B) Representative γH2AX (red) staining of a mitotic cell in E7 retina at 3 hrs after 2 Gy irradiation. Nuclei were counterstained with DAPI (blue). Note that cells that enter mitosis 3 hrs after the treatment still harbor unrepaired DSBs. Scale bar = 2 μm.
