# Supplementary material for: Cell Cycle Regulation and Apoptotic Responses of the Embryonic Chick Retina by Ionizing Radiation
Source: PLoS One. 2016 May 10;11(5):e0155093. doi: 10.1371/journal.pone.0155093 (PMC4862647; doi:10.1371/journal.pone.0155093)
Supplement: S6 Fig — (A-H) cc3 staining (green) in control and 2 Gy irradiated E3 retinae at 3, 6, 12 and 24 hrs after treatment. Nuclei were counterstained with DAPI (blue). Note highest abundance of apoptotic cells at 12 hrs after treatment. (I-J) Quantification of cc3+ cells (green, encircled in K) 12 hrs after irradiation with 0.5, 1 and 2 Gy in central parts of E3 retinae. Note strong increase of apoptotic events after dose doubling from 0.5 to 1, but no further increase after doubling the dose from 1–2 Gy. Data are presented as means (n = 3, with at least four different pictures analyzed for each experiment) ± SEM (*P<0.05 **P< 0.01 *** P<0.001). Scale bar = 25 μm. RPE, retinal pigmented epithelium; pONL presumptive outer nuclear layer. (PDF) [file pone.0155093.s006.pdf]

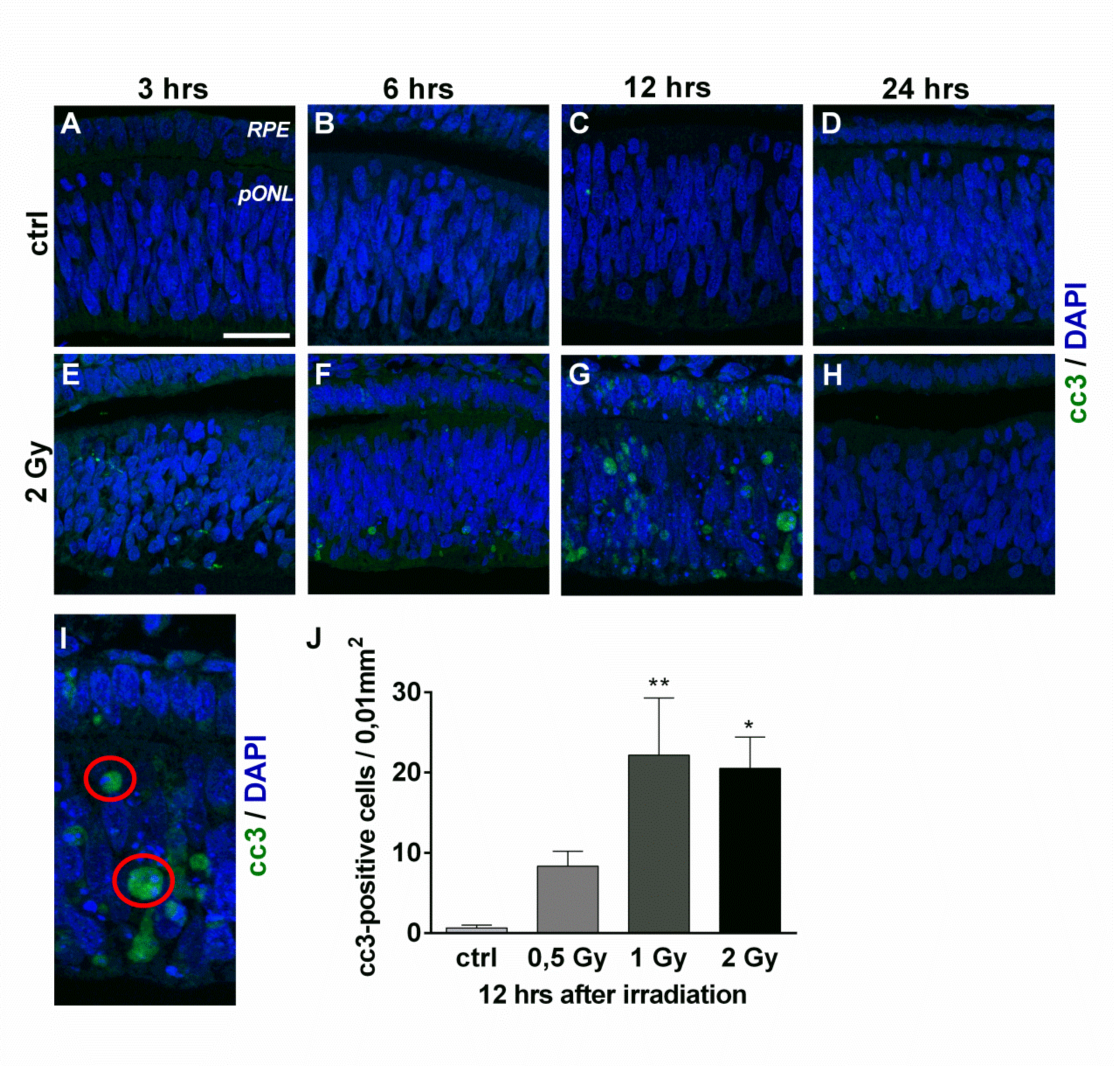

S6 Fig. Radiation induced apoptosis peaks at 12 hrs after irradiation in E3 retina. (A-H) cc3 staining (green) in control and 2 Gy irradiated E3 retinae at 3, 6, 12 and 24 hrs after treatment. Nuclei were counterstained with DAPI (blue). Note highest abundance of apoptotic cells at 12 hrs after treatment. (I-J) Quantification of $cc3^{+}$ cells (green, encircled in K) 12 hrs after irradiation with 0.5, 1 and 2 Gy in central parts of E3 retinae. Note strong increase of apoptotic events after dose doubling from 0.5 to 1, but no further increase after doubling the dose from 1-2 Gy. Data are presented as means (n = 3, with at least four different pictures analyzed for each experiment) ± SEM (*P<0.05 **P< 0.01 *** P<0.001). Scale bar = 25 μm. RPE, retinal pigmented epithelium; pONL presumptive outer nuclear layer.
