# Supplementary material for: Cell Cycle Regulation and Apoptotic Responses of the Embryonic Chick Retina by Ionizing Radiation
Source: PLoS One. 2016 May 10;11(5):e0155093. doi: 10.1371/journal.pone.0155093 (PMC4862647; doi:10.1371/journal.pone.0155093)
Supplement: S7 Fig — (A-I) cc3 staining (green) in control, 1 and 2 Gy irradiated E5 retinae at 12 hrs after treatment. Nuclei were counterstained with DAPI (blue). Note absence of cc3+ cells in central retina, and also their strong increase from 1 to 2 Gy in intermediate and central parts of the retina. (J) Quantification of cc3+ cells in central E5 retina at defined time points after irradiation with 1 and 2 Gy. RIA peak 12 hrs after irradiation; note correlation between doses and RIA, and also between appearance of cc3+ and pyknotic nuclei. Data are presented as means (n = 2) ± SEM (*** P<0.001). Scale bar = 50 μm. RPE, retinal pigmented epithelium; pONL, presumptive outer nuclear layer. (PDF) [file pone.0155093.s007.pdf]

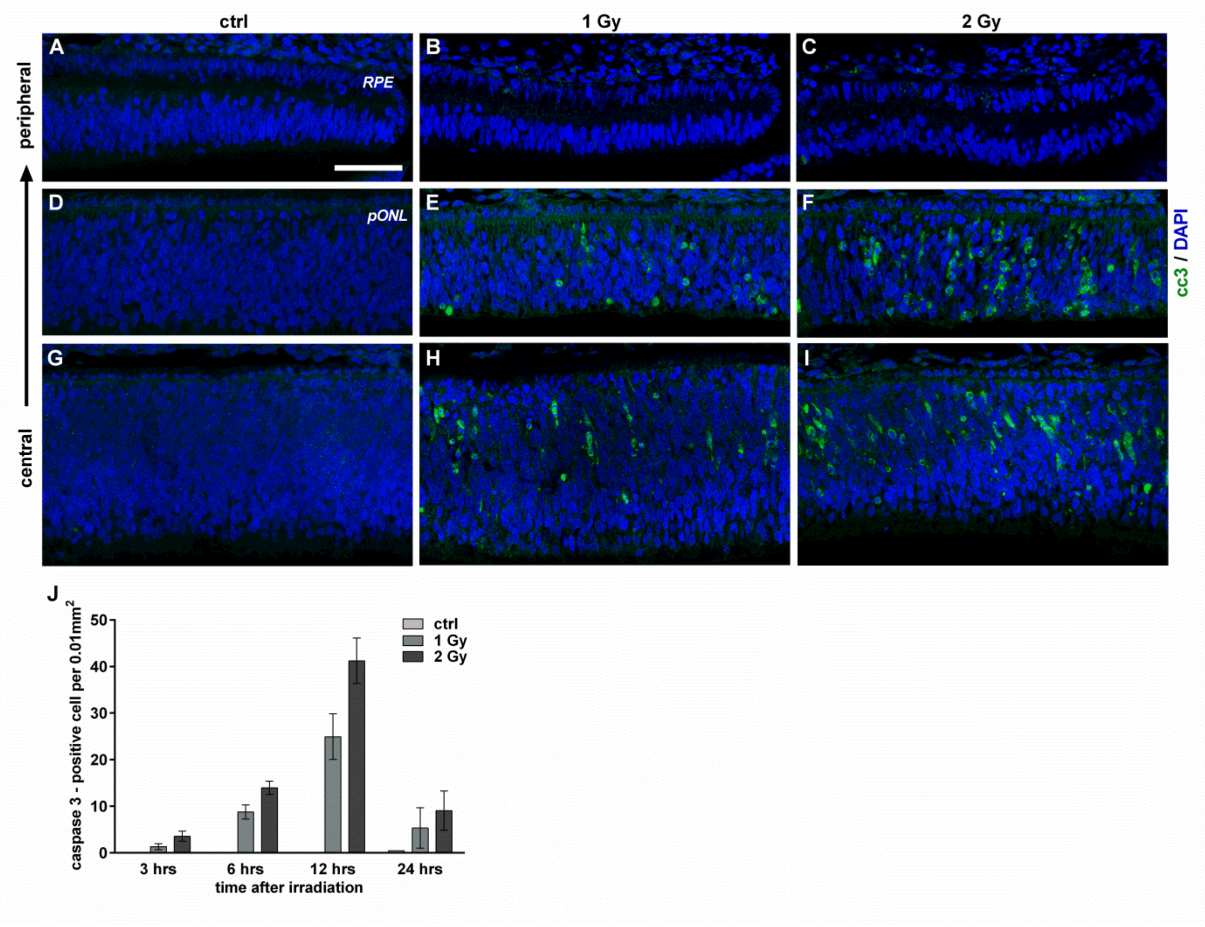

S7 Fig. Radiation induced apoptosis peaks at 12 hrs after irradiation in E5 retina. (A-I) cc3 staining (green) in control, 1 and 2 Gy irradiated E5 retinae at 12 hrs after treatment. Nuclei were counterstained with DAPI (blue). Note absence of $cc3^{+}$ cells in central retina, and also their strong increase from 1 to 2 Gy in intermediate and central parts of the retina. (J) Quantification of cc3+ cells in central E5 retina at defined time points after irradiation with 1 and 2 Gy. RIA peak 12 hrs after irradiation; note correlation between doses and RIA, and also between appearance of cc3+ and pyknotic nuclei. Data are presented as means (n = 2) ± SEM (*** P<0.001). Scale bar = 50 µm. RPE, retinal pigmented epithelium; pONL, presumptive outer nuclear layer.
