# Supplementary material for: Cell Cycle Regulation and Apoptotic Responses of the Embryonic Chick Retina by Ionizing Radiation
Source: PLoS One. 2016 May 10;11(5):e0155093. doi: 10.1371/journal.pone.0155093 (PMC4862647; doi:10.1371/journal.pone.0155093)
Supplement: S8 Fig — (A-I) cc3 staining (green) in control, 1 and 2 Gy irradiated E7 retinae at 3 hrs after treatment. Nuclei were counterstained with DAPI (blue). Note absence of RIA after 1 Gy, and—after 2 Gy (C, F, I)—higher numbers in peripheral than in central parts of the retina. (J) Determination of cc3 activity in lysates of whole E7 retinae from controls and embryos irradiated with 2 Gy at defined time points after treatment. Data are presented as means (n = 3) ± SEM (*** P<0.001). Scale bar = 50 μm. RPE, retinal pigmented epithelium; pONL, presumptive outer nuclear layer. (PDF) [file pone.0155093.s008.pdf]

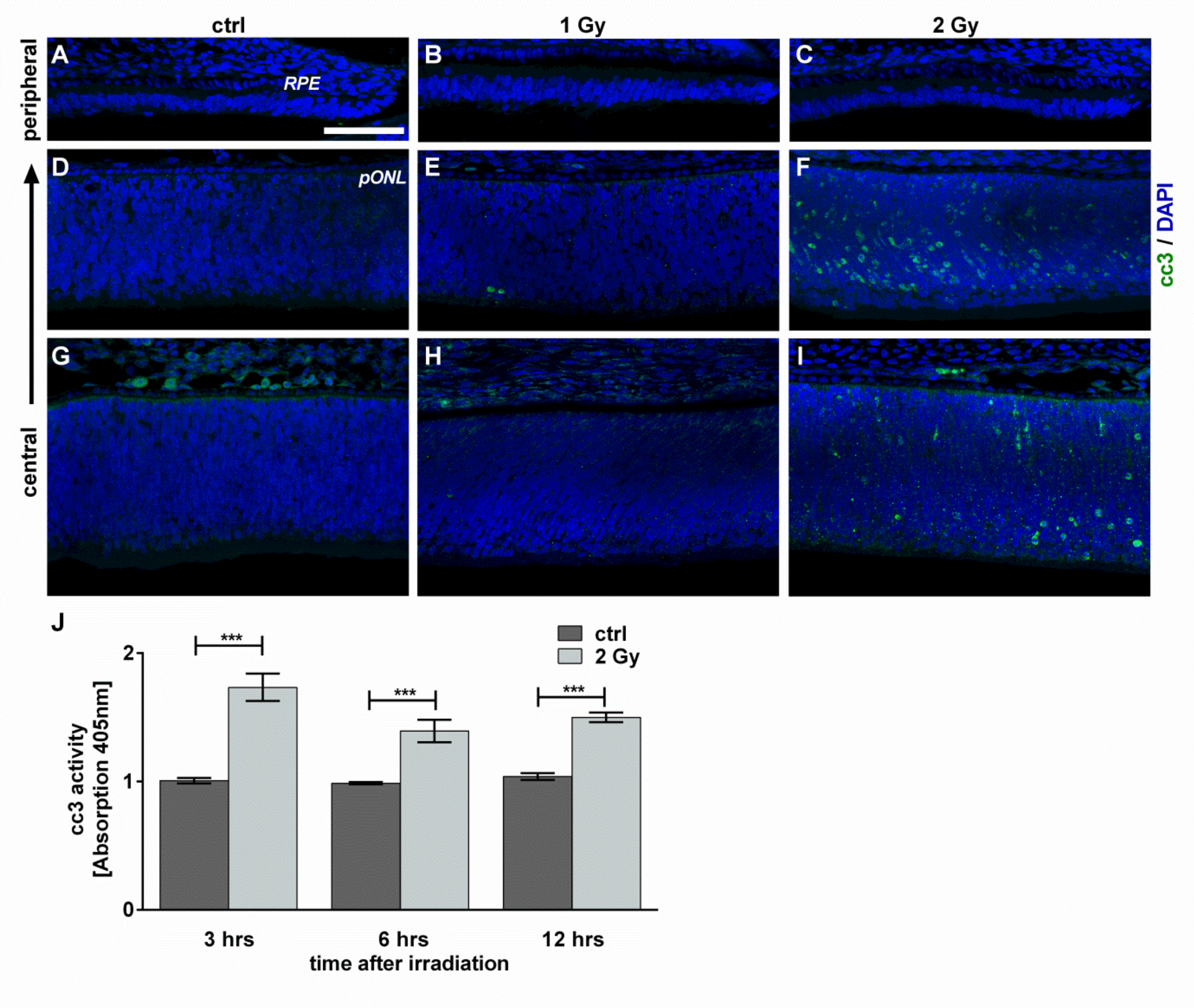

S8 Fig. Radiation induced apoptosis peaks at 3 hrs after irradiation in E7 retina. (A-I) cc3 staining (green) in control, 1 and 2 Gy irradiated E7 retinae at 3 hrs after treatment. Nuclei were counterstained with DAPI (blue). Note absence of RIA after 1 Gy, and - after 2 Gy (C, F, I) - higher numbers in peripheral than in central parts of the retina. (J) Determination of cc3 activity in lysates of whole E7 retinae from controls and embryos irradiated with 2 Gy at defined time points after treatment. Data are presented as means (n = 3) ± SEM (*** P<0.001). Scale bar = 50 µm. RPE, retinal pigmented epithelium; pONL, presumptive outer nuclear layer.
