# Supplementary material for: Cell Cycle Regulation and Apoptotic Responses of the Embryonic Chick Retina by Ionizing Radiation
Source: PLoS One. 2016 May 10;11(5):e0155093. doi: 10.1371/journal.pone.0155093 (PMC4862647; doi:10.1371/journal.pone.0155093)
Supplement: S9 Fig — (PDF) [file pone.0155093.s009.pdf]

Supplemental Experimental Procedures

Assay for cleaved caspase-3 activity

For measurements of cleaved caspase 3 (cc3) activity after *in ovo* irradiation, retinae were isolated at defined time points after irradiation, washed in PBS and snap-frozen in liquid nitrogen. The caspase-3 cell assay (BML-AK703, Enzo) was performed according to the manufacturer's protocol. Absorption was measured at 405 nm.

BrdU labeling for Rad51 staining

Eggs from the white leghorn chick (Gallus gallus domesticus) were incubated at 37°C and 65% humidity for 2 days. Then a window was cut into the egg shell for Hamburger Hamilton (HH) stage determination and BrdU application. Before treatment at E7, HH stage was determined (HH 30-32). Irradiation was performed with 135 kV and 19 mA, using a Philips MCN 165/796704 X-Ray machine, equipped with a Tungsten anode and a Beryllium window with a dose of 2 Gy, as estimated by using an ion chamber (PTW) that was placed on top of the egg; the dose rate was 0.5 Gy / min. 100 µl of a 25 mM BrdU solution (Boehringer, solved in PBS) were pipetted on the top of the embryo directly after irradiation. Fixation was done 1 h after irradiation. For tissue isolation, eyes were collected in F-12 medium (Gibco) on ice. For immunohistochemistry eyes were fixed in PBS containing 4% formalin (pH 7.3) for 16 hrs. Formalin-fixed tissues were embedded in paraffin and sectioned at a thickness of 3 µm.
